# The Power of Access in Parkinson's Disease Care: A Retrospective Review of Telehealth Uptake During the COVID-19 Pandemic

**DOI:** 10.3389/fneur.2022.830196

**Published:** 2022-04-07

**Authors:** Drew Falconer, Sonia Gow, David Whitney, Hannah Walters, Sean Rogers

**Affiliations:** Inova Parkinson's and Movement Disorders Center, Falls Church, VA, United States

**Keywords:** telehealth, Parkinson's disease, movement disorders, specialty care access, DBS (deep brain stimulation), telemedicine (keywords), patient access, access to care

## Abstract

**Objective:**

The onset of the COVID-19 pandemic in March of 2020 forced a rapid pivot to telehealth and compelled a use-case experiment in specialty telehealth neurology movement disorders care. The aims of this study were to quantify the potential benefit of telehealth as an option to the Parkinson's disease community as shown by the first 9 months of the COVID-19 pandemic, and to quantify the potential impact of the absence of a deep brain stimulation (DBS) telehealth option on DBS patient follow-up.

**Methods:**

New patient visits to the Inova Parkinson's and Movement Disorder's Center from April to December 2020 (9 months) were retrospectively reviewed for telehealth vs. in-person, demographics (age, gender, race, primary insurance), chief complaint, prior movement disorders specialist (MDS) consultation, imaging tests ordered, and distance/travel time from primary zip code to clinic. Additionally, DBS programming visit volume from April to December 2020 was compared to DBS programming visit volume from April to December 2019.

**Results:**

Of the 1,097 new patients seen, 85% were via telehealth (*N* = 932) and 15% in person (*N* = 165). In the telehealth cohort, 97.75% had not consulted with an MDS before (*N* = 911), vs. 87.9% of in-person (*N* = 145). Age range was 61.8 +/– 17.9 years (telehealth), 68.8 +/– 16.0 years (in-person). Racial breakdown for telehealth was 60.7% White (*N* = 566), 10.4% Black (*N* = 97), 7.4% Asian (*N* = 69) and 4.5% Hispanic (*N* = 42); in-person was 70.9% White (*N* = 117), 5.5% Black (*N* = 9), 7.9% Asian (*N* = 13) and 5.5% Hispanic (*N* = 9). Top 5 consultation reasons, top 10 primary insurance providers and imaging studies ordered between the two cohorts were similar. Distance/travel time between primary zip code and clinic were 33.8 +/– 104.8 miles and 42.2 +/– 93.4 min (telehealth) vs. 38.1 +/– 114.7 miles and 44.1 +/– 97.6 min (in-person). DBS programming visits dropped 24.8% compared to the same period the year before (254 visits to 191 visits).

**Conclusion:**

Telehealth-based new patient visits to a Movement Disorders Center appeared successful at increasing access to specialty care. The minimal difference in supporting data highlights the potential parity to in-person visits. With no telehealth option for DBS visits, a significant drop-off was seen in routine DBS management.

## Introduction

With the discovery and rapid proliferation of the coronavirus SARS-Cov-2 (COVID-19) in early 2020, medical care as a whole shifted rapidly to meet a changing landscape of patient needs. Inpatient and hospital-based clinical teams adapted to new safety requirements and an increase in both patient volume and acuity. At the same time, most outpatient clinics pivoted quickly to integrate a telehealth-based option into their workflow, balancing safety with access and continuity of care (1). This rapid change in the delivery of outpatient healthcare resulted in a shift whereby within a few weeks, the adoption of telehealth offset two-thirds of the decline in in-person clinical visits (2).

Prior to 2020, telehealth was viewed as a challenge for many older patients. In a 2018 study, 80% of older patients queried could successfully complete a telephone visit yet 38% reported being unable to successfully connect to a video visit (3). Reasons for this were broad and included such concerns as comfort with technology, physical or cognitive disability, privacy and IT security, telehealth platform design, internet connections and cost (3, 4). That said, the benefits of telehealth for access to care were already being established across medical disciplines, especially regarding the management of chronic conditions (5–9). Adoption of technology was also increasing in the 65 years and older population. One pre-pandemic Pew Research study reported roughly two-thirds of persons 65 years and older interacting with the internet, and smart phone ownership quadrupling in that age group in only 5 years. However, the same survey showed that 73% of persons over 65 reported needing help to set up or use a new device (10), thus reflecting increased access to technology but perhaps not a high level of comfort with those devices.

With the onset of the COVID-19 pandemic, the need for telehealth was no more acute than in Neurology clinics, specifically amongst Parkinson's and Movement Disorders specialty clinics. These patients are generally over the age of 65 and have chronic medical illnesses, putting them at a higher risk of hospitalization and poor outcomes from COVID-19 infection (11, 12). Furthermore, with the natural progression of Parkinson's disease (PD) and the absolute need for both longitudinal care and rehab services, delaying care due to poor clinical access or deferral of care had the potential to significantly set back the motor and non-motor function of many patients (12–15).

Prior studies had already established the viability of telehealth visits for Parkinson's disease patients, demonstrating positive patient and provider satisfaction as well as significant travel and cost savings for patients but no identifiable drop-off in quality of care nor outcomes (1, 16–19). One study showed that after completing a successful telehealth clinical visit, 80% of PD patients reported willingness to use telehealth again given the benefits of reduced travel time and improved access (20). This highlighted some of the known limitations of in-person specialty Parkinson's and Movement Disorders care: limited access, onerous burdens of the time and physical act of travel, as well as for many, the logistical challenges needed to schedule a clinical visit, travel to the visit then return home (19–21). Once the COVID-19 pandemic began, this was compounded by social limitations as well as patient's fears regarding the perceived safety of medical care and travel (22).

Born of necessity and with the above issues in mind, the Inova Parkinson's and Movement Disorders Center (IPMDC) pivoted quickly to offer telehealth visits to both new and follow-up patients starting in mid-March 2020. In-person visits were limited to a certain number per day, and initially only offered to patients requiring in-person procedures such as botulinum toxin injections and DBS adjustments. Very few clinical encounters were allowed to be scheduled face-to-face during the first few months of the pandemic due to local and national stay at home orders, limited to only very specific circumstances. Despite this the goals of clinical care remained unchanged: maintain patient-provider access to best manage the changes of a chronic, progressive medical condition, while navigating the disruption of the global pandemic.

IPMDC is a community-based Parkinson's and Movement Disorders Center, built within the integrated health network of the Inova Health System in Northern Virginia. At the time of the COVID-19 pandemic's onset, IPMDC was home to three fellowship-trained Parkinson's and Movement Disorders specialists running clinical care five days a week and in doing so, caring for a large and growing community of Parkinson's and other movement disorder patients.

This retrospective chart review study came about after the rapid and surprising uptake of new patient clinical appointments made after March 2020 to the IPMDC, where between the months of April and December, 1,097 new patients were evaluated with what seemed to be the vast majority having never consulted with an MDS before. Additionally, a drop-off of DBS follow-up visits was observed by the clinical team, presumably due to the necessity for an in-person visit to adjust the DBS system. Offering telehealth-based new patient appointments seemed to make engaging with an MDS possible for some who before believed it was not logistically an option, while the lack of a telehealth option appeared to limit access to a procedure-based clinical encounter such as a DBS programming. This is all within the context of the obvious early bias toward virtual visits during the beginning of the pandemic. Regardless, these circumstances allowed for a rapid test-case for offering telehealth services, and thus this retrospective chart review of the new patient appointments for the first nine months of the COVID-19 pandemic was done in an attempt to quantify the impact of telehealth on access to specialty care MDS.

## Materials and Methods

De-identified data was retrospectively collected from the 1,097 new patients seen by the IPMDC from April 1, 2020 to December 31, 2020 (first 9 months of the COVID-19 pandemic). A comparative univariate and multivariate analysis were then applied to the data using SAS statistical software. New patients aged 18 to 98 years old were included for analysis. Exclusion criteria were patient visits designated follow-up visits and visits completed before April 1, 2020 or after December 31, 2020.

The primary objectives were to gain a better picture of the degree of PD patients who gained first-time access to specialty care via the utilization of telehealth, compare the average distance which would have been traveled if the visits were in-person instead of telehealth, determine the degree of ancillary testing ordered via telehealth vs. in-person visits, and more. In collecting the data, there were charts which did not include one of the metrics identified; they were excluded from the calculation of that metric.

The secondary objective was to compare the frequency of DBS programming visits during the first 9 months of the COVID-19 pandemic with the same timeframe the year prior, with the goal of quantifying the potential impact of the absence of a telehealth option on the availability of clinical encounters for the DBS population.

The following data points were collected under a randomized patient identifier:

- Age
- Gender
- Race
- Zip code of patient's primary address
- Primary insurance provider for the patient
- Movement Disorders Specialist seen
- New patient visit date
- Visit type (telehealth or in-person)
- History of prior MDS consultation
- Primary reason for consultation
- Imaging tests ordered at this visit (MRI, CT, DaTscan)

Once collected, an Excel Driving Distance Calculator was used through Google Analytics to calculate the driving distance and low traffic travel time from the subject's primary zip code to the primary IPMDC clinic in Alexandria, VA.

A comparative univariate and multivariate analysis was then applied to some data using SAS statistical software, while others were presented as a simple comparison with percentages.

Regarding the secondary objective, the frequency of DBS programming visits during the first 9 months of the COVID-19 pandemic was compared with the same timeframe the year prior, with the goal of quantifying the potential impact of the absence of a telehealth option on the availability of clinical encounters for the DBS population. During the first nine months of the COVID-19 pandemic, DBS programming visits necessitated an in-person encounter. For this outcome measure, the inclusion criteria were age 18 to 98 years old and being designated a DBS programming visit (new and follow-up) conducted from April 1, 2020 to December 31, 2020. This was compared to a pre-pandemic cohort of DBS programming visits (new and follow-up) which were conducted from April 1, 2019 to December 31, 2019. To identify these patients, a deidentified count was made of clinical visits where the CPT code 95983 (denoting the first 15 min of DBS programming) was used during the timeframes above.

Telehealth visits were completed between the patient and the provider through Zoom, Doximity or Vidyo applications. A waiver of informed consent and a waiver of HIPAA authorization was granted for this retrospective chart review study.

## Results

During the first 9 months of the COVID-19 pandemic between April 1, 2020 and December 31, 2020, the Movement Disorders Specialists at the Inova Parkinson's and Movement Disorders Center saw 1,097 new patients. Of these new patients, 85% were conducted via a telehealth platform (*N* = 932), and 15% were conducted in-person (*N* = 165).

Only 2.25% of the telehealth-based visit cohort were documented to have seen an MDS before (*N* = 21), meaning 97.75% of the new patient telehealth-based visit cohort had never consulted with a specialist before (*N* = 911). When comparing this to the in-person new patient visit cohort, 12.1% were documented as having consulted with an MDS before (*N* = 20), with 87.9% having never consulted with a specialist before (*N* = 145) (*P* < 0.0001) (Table 1).

**Table 1:** Characteristics of virtual and in-person cohorts.

	**Virtual (*****N*** **= 932)**		**In-person** **(*****N*** **= 165)**		
**Variable**	**-**	**[min-max]**	**-**	**[min-max]**	***p*** **-value**
Male	475 (51.0%)		78 (47.3%)		0.399
Age	61.8 ± 17.9 (919)	[18–98]	66.8 ± 16.0 (165)	[18–92]	<0.001
Travel time (min)	42.2 ± 93.4 (923)	[5–1881]	44.1 ± 97.6 (165)	[5–1242]	0.812
Distance to Clinic (miles)	33.8 ± 104.8 (923)	[1.49–2120.65]	38.1 ± 114.7 (165)	[1.49–1406.71]	0.629
CT	19 (2.0%)		1 (0.6% )		0.341
MRI	221 (23.7%)		30 (18.2%)		0.132
DatScan	61 (6.5%)		11 (6.7%)		1.000
Seen MDS Before	21 (2.3%)		20 (12.1%)		<0.001
White	566 (60.7%)		117 (70.9%)		0.015
Black	97 (10.4%)		9 (5.5%)		0.046
Asian	69 (7.4%)		13 (7.9%)		0.872
Hispanic	42 (4.5%)		9 (5.5%)		0.550

When noting the primary reasons for consultation with the IPMDC, the top diagnoses in both groups outside of not listed, were Tremor (24.8% of in-person vs. 21.6% of telehealth), Parkinson's disease (15.2% of in-person vs. 16.6% of telehealth), Memory Loss (6.7% of in-person vs. 7.5% of telehealth), Stroke (4.2% of in-person vs. 5.9% of telehealth), and Numbness (5.5% of in-person vs. 4.4% of telehealth). A proportion of new patient visits did not have a reason for referral or active referral form documented. As they were seen in a Parkinson's and Movement Disorders Center, the presumption is that most of those referrals were for MDS evaluation (Table 2).

**Table 2:** Most common 5 diagnosis in both cohorts.

	**Virtual**		**In-Person**	
**Ranking**	**Diagnosis**	**N (%)**	**Diagnosis**	**N (%)**
1	Tremor	201 (21.6%)	Tremor	41 (24.8%)
2	Parkinson's	155 (16.6%)	Parkinson's	25 (15.2%)
3	Memory Loss	70 (7.5%)	Memory Loss	11 (6.7%)
4	Stroke	55 (5.9%)	Stroke	7 (4.2%)
5	Numbness	41 (4.4%)	Numbness	9 (5.5%)

### Demographics and Insurance Coverage

Comparing the demographic breakdown of both cohorts, the telehealth-based cohort was 51% male (*N* = 475) while the in-person cohort was 47.3% male (*N* = 78) (*P* 0.3991). The average age for the telehealth-based cohort was 61.8 +/– 17.9 years (range 18 to 98 years old), while average age for the in-person cohort was 68.8 +/– 16.0 years (range 18 to 92 years) (*P* 0.0008) (Table 1).

Self-identified racial breakdown of the telehealth-based cohort were 60.7% White (*N* = 566), 10.4% Black (*N* = 97), 7.4% Asian (*N* = 69) and 4.5% Hispanic (*N* = 42). The in-person cohort was 70.9% White (*N* = 117), 5.5% Black (*N* = 9), 7.9% Asian (*N* = 13) and 5.5% Hispanic (*N* = 9). These top 4 racial designations accounted for 83.0% of the new patient telehealth-based visits (*N* = 774) and 89.7% of the new in-person visits (*N* = 148) (Table 1).

The top insurance provider in both cohorts was Medicare and Medicare MCO, accounting for a combined 41% of visits (39.5% of in-person vs. 50.9% of virtual). The next most common primary insurance providers for both cohorts were Medicaid and then Federal Blue Cross/Blue Shield (common in our area given the IPMDC's proximity to Washington, DC). All four of the top insurance plans are considered federal plans, and thus federal, non-private insurance plans make up 63.6% of the in-person new consultations and 57.3% of the virtual new consultations (Table 3).

**Table 3:** Payer by visit type.

	**Virtual**	**In-person**
**Primary payer**	**N (%)**	**N (%)**
MediCare	270 (29.0%)	65 (39.4%)
Medicare MCO	98 (10.5%)	19 (11.5%)
Medicaid HMO	85 (9.1%)	14 (8.5%)
FEP BCBS	81 (8.7%)	7 (4.2%)
United Healthcare	64 (6.9%)	8 (4.8%)
CIGNA	62 (6.7%)	12 (7.3%)
AETNA	61 (6.5%)	11 (6.7%)
N/A	52 (5.6%)	4 (2.4%)
Anthem	46 (4.9%)	7 (4.2%)
Carefirst	45 (4.8%)	8 (4.8%)

### Imaging Tests, Distance Traveled and Volume Change Over Time

Comparing the imaging tests ordered during the new patient visit, more CT scans were ordered virtually (0.6% of in-person vs. 2.0% of telehealth, *P* 0.3415), slightly more MRI scans were ordered via telehealth (18.2% of in-person vs. 23.7% of telehealth, *P* 0.1317), and approximately the same number of DaTscan PET imaging were ordered (6.7% of in-person vs. 6.5% of telehealth, *P* 1.00) (Table 4).

**Table 4:** Imaging volume by visit type.

	**Virtual**	**In-person**
**Imaging**	**N (%)**	**N (%)**
CT	19 (2.0%)	1 (0.6%)
MRI	221 (23.7%)	30 (18.2%)
DatScan	61 (6.5%)	11 (6.7%)

Average driving distance that would have been traveled by the telehealth cohort (33.8 +/– 104.8 miles) was approximately the same as the distance traveled by the in-person cohort (38.1 +/– 114.7 miles), (*P* 0.6287). Low-traffic travel time was approximately the same, with the travel time of the telehealth cohort 42.2 +/– 93.4 min and the travel time of the in-person cohort 44.1 +/– 97.6 min (*P* 0.8117) (Table 1).

**Figure 1:**
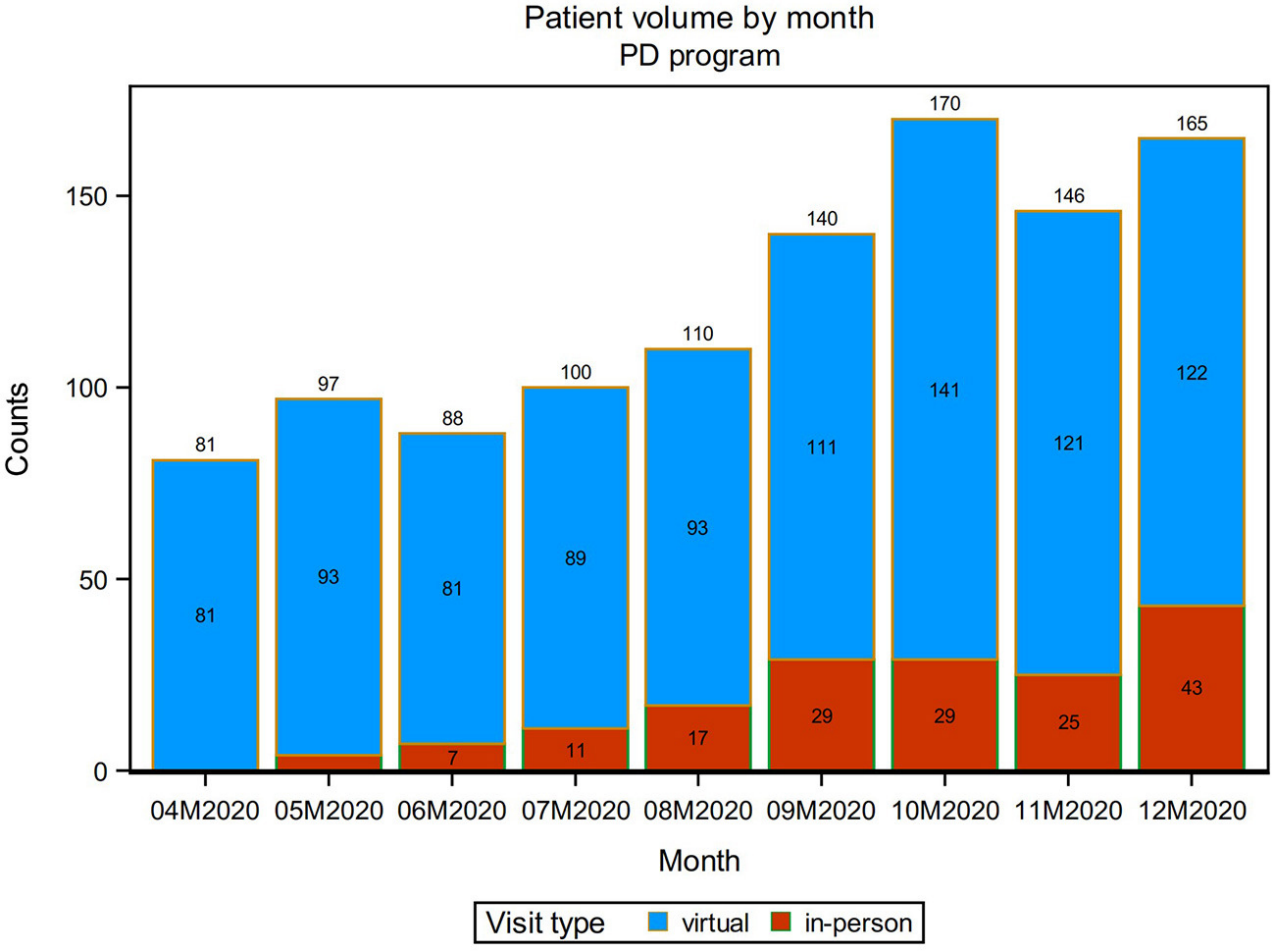
Change in visit type from April 2020 (04M2020) to December 2020 (12M2020).

Over the course of the first 9 months of the pandemic from April 1, 2020 to December 31, 2020, the overall new patient volume (in-person and virtual) increased steadily from 81 new patients seen in April 2020 to a maximum of 170 new patients seen in October 2020. The number of patients who were seen in-person also increased steadily over the first 9 months of the pandemic, from 0 of the new patients seen in April 2020 to reaching its maximum of 43 in December 2020. Throughout, the majority of new patient visits were completed via telehealth, at minimum 81 a month and at maximum 141 a month (Figure 1).

### Change in Face-to-Face DBS Programming Visits

DBS programming required an in-person encounter. Total IPMDC visits using CPT code 95983 from April 1, 2020 to December 31, 2020 were compared to the same visit type from April 1, 2019 to December 31, 2019. During the timeframe of April 1, 2019 to December 31, 2019, 254 such visits were conducted. During the same timeframe in 2020, denoting the first 9 months of the COVID-19 pandemic, 191 DBS programming visits were conducted, reflecting a 24.8% drop in DBS programming visits compared to the prior year (Table 5).

**Table 5:** DBS clinical programming volume from April 1, 2019 to December 31, 2019 compared to April 1, 2020 to December 31, 2020.

**Time frame**	**N**
April 2019 to December 2019	254
April 2020 to December 2020	191

## Discussion

Prior to the onset of the COVID-19 pandemic in 2020, a discussion was already beginning within the movement disorders specialty regarding improving access for underserved populations using telehealth (1, 16–19). For years, the statistic that as few as 28% of Parkinson's disease patients were seeing an MDS has been seen as one of the many hurdles limiting the utilization of newly FDA-approved treatments (23).

Overall, neurology as a field suffers from variable density of neurologists throughout the US, and this access issue is enhanced when considering fellowship trained movement disorders specialists. One recent study showed that ~20% of adult Medicare patients traveled outside of their hospital referral region for care with an average distance traveled of 148.7 miles. In this study, the most common neurological condition among patients who traveled outside of their home region was Parkinson's disease (24). That reflects only those patients who are able to travel. The limits of distance, logistics of travel, and time as well as physical and cognitive limitations keep many patients from seeking the highest level of care for their movement disorders diagnosis (25). These hurdles do not take into account the challenge of making a clinical appointment, even if travel were not an issue. On average in the U.S. wait time for a new patient visit with a Movement Disorder Specialist (MDS) is 2.2 months with a range of 2 to 8 months. Half of U.S. MDS Centers report a wait time longer than 2 months and approximately one-third of U.S. centers report wait times > 3 months (26). Without alternative solutions such as digital/telehealth options, many patients were more likely to delay or forego much needed care or simply believed that specialty care was unobtainable (22).

The onset of the COVID-19 pandemic in March of 2020 forced a rapid pivot to telehealth across the US, and at the same time, compelled a use-case experiment in specialty telehealth movement disorders care. At the IPMDC, this resulted in a significant increase in new patient visits starting in April of 2020 and continuing through at least the end of that year. Most new patient visits were completed via telehealth (85%) and the vast majority of those patients had never consulted with a specialty care MDS before (97.75%). This demonstrates how the offering of telehealth new patient visits created an opportunity for patients who before would not have been able to manage the logistics of an in-person visit related to mobility, distance, travel, and time. The increased uptake also suggests the upward trend of technology adoption in the 65-year-old-plus population likely also accelerated, as the number of new patient visits to the IPMDC via telehealth also increased over the first 9 months of the pandemic, hitting a high of 141 in October 2020.

There was a statistically significant difference in the average age of the two cohorts (61.8 years old virtual vs. 66 years old in-person, *P* = 0.0008), but not a significant difference in the travel time or distance between the patient and clinic (33.8 miles and 42.2 min virtually vs. 38.1 miles and 44.1 min in-person). This appears to highlight the universal utilization of telehealth visits across the age and distance spectrum, and the ability for those outside of the expected groups to capitalize on the logistical ease inherent to a telehealth visit. While telehealth visits may be most obviously beneficial for nursing home or assisted living patients, perhaps this shows an equal utility among younger patients who find it more convenient to log on to a virtual visit instead of taking significant time off work for an in-person visit. Or perhaps in general even those patients who could make an in-person visit simply preferred telehealth. This was exemplified by the continued high proportion of telehealth visits completed later in 2020 when in-person visits were more widely available. The universal appeal was also shown in the similarity in reasons for referral between the two cohorts, as the top 5 diagnoses offered were the same, reflecting little favoring of one diagnosis over another regarding a telehealth option. All of these factors reflect an increase in access to specialty Movement Disorders care—be it a prior limitation of distance or simply the logistics of a clinical visit irrespective of distance.

Most would expect a higher reliance on neuroimaging as a supplement to a reduced physical examination via a telehealth platform, and this data does show a higher rate of CT scans ordered via the telehealth cohort (2.0% vs. 0.6%), though noting the rare use of CT scan overall. MRI scans were ordered a slightly higher rate via telehealth at 23.7% vs. 18.2% and DaTscan PET imaging was also ordered at approximately the same rate between the two cohorts (6.5% telehealth vs. 6.7% in-person). This refutes the notion that telehealth necessitates higher reliance on neuroimaging, and in fact points toward relative parity between the workups initiated in-person vs. through a telehealth platform.

When evaluating the racial breakdown of the two cohorts, there appears to be little difference between the telehealth and in-person utilization of patients identifying as Asian or Hispanic, but those identifying as Black made up twice the percentage of virtual visits (10.4%) vs. in-person (5.5%). Black patients represent a traditionally underserved community within specialty Parkinson's disease care due to complex issues related to economic resources and insurance status as well as multifaceted organizational and social/cultural barriers (27). This two-fold increase in new patient visit utilization by Black patients suggests that telehealth may help alleviate some of the perceived barriers to seeking specialty care.

Regarding primary insurance coverage, the top 10 insurance providers were the same when comparing virtual new patient visits vs. in-person, with the top three in each Medicare/Medicare MCO, then Medicaid, then Federal Blue Cross/Blue Shield. When comparing the two groups, there was a 10% higher relative utilization of in-person visits compared to virtual visits within the Medicaire/Medicare MCO group (39.5% virtual vs. 50.9% in-person), but Federal BCBS patients favored telehealth by about two to one (8.7% vs. 4.2%). Perhaps this represents the preference for an in-person new patient visit for those over 65 who have Medicare, though noting the approximately 6 year average age difference between the two cohorts (61.8 +/– 17.9 years old telehealth vs. 68.8 +/– 16.0 years old in person).

When considering DBS patient visits, which prior to 2021 required an in-person visit to interrogate and program the DBS device, a 2020 study showed that 77% of DBS patients rely on another person for transport and 79% of DBS patients surveyed would see a more experienced DBS doctor, even out of state, if that doctor offered telehealth (28). This takes on different context when the median distance traveled to the nearest Movement Disorders specialty center for all patients is 56.1 miles, and even further for those in need to DBS management at 87.5 miles (29). Given the lack of a reliable telehealth-based DBS programming option in 2020, it comes as no surprise that the DBS programming visit volume at the IPMDC dropped by 24.8% compared to the same timeframe in 2019 (254 visits to 191 visits). Those that could forego their DBS adjustments did so during the first peak of the pandemic and *de novo* DBS implants were postponed in line with the early pandemic canceling of elective surgical cases. Ongoing studies related to telehealth DBS services, now FDA approved, will give a better picture of the utilization of a DBS telehealth option.

While this study suggests the benefits of telehealth regarding access, it bears noting the continued hurdles related to telehealth experienced by many. This includes access to reliable internet and technology, technical limitations of both hardware and software use, as well as for many the need for a care partner to successfully connect and complete a telehealth visit.

Finally, this data cannot be considered without pointing out the extenuating circumstances and limitations that were present regarding health market dynamics in the first nine months of the COVID-19 pandemic. At the beginning, all patients were shifted to a telehealth model or asked to delay their care. Though this mandate was loosened as the year went on, many patients continued to choose a telehealth option out of concern for safety as well as ease of access. While this is a known conflicting factor, future studies will hopefully help to quantify the impact that necessity had on telehealth uptake and help to delineate the role of telehealth on a potential volume and population-based increase in specialty care access. Additionally, the ideal metric on which to measure this data would be a comparison to pre-pandemic trends and percentage. This would represent a significant additional chart review which can be done in a follow-up study and was not possible within the framework and time dedicated to this study.

## Conclusion

At the Inova Parkinson's and Movement Disorders Center, the forced experiment of telehealth new patient visits during the first 9 months of the COVID-19 pandemic was by all measures a success. Being able to reach MDS providers virtually without the logistical and physical hurdles of an in-person visit allowed 911 of the telehealth-based new patients to consult with an MDS for the first time, representing 97.75% of the new telehealth-based patients. Additionally, the telehealth option resulted in twice as many Black new patient consults by percentage, possibly reflecting an avenue for increased access for a traditionally underserved community. Given the absence of a telehealth option for DBS programming visits, a significant drop-off (24.8%) was seen in visits involving routine DBS device management compared to the same timeframe in the pre-pandemic year before.

The minimal differences in age, gender, travel time and distance, chief complaint and imaging test utilization highlight the seemingly universal appeal of telehealth specialty services beyond simply the high-acuity and limited mobility patients. As more and more studies are published involving the parity of care and outcomes delivered via a telehealth model vs. a traditional in-person visit, this data aids that discussion, and suggests that the question should not be either/or, but simply how telehealth can continue to be an option that empowers patients with the benefits of moving beyond the hurdles of distance, travel, and time. If telehealth allows for greater and easier access to care of any type, including specialty care, and the percentage of Parkinson's patients able to partner with an MDS climbs beyond the current 28%, then the opportunity born out of a tragic scenario will have elevated our profession as a whole.

## Data Availability Statement

The raw data supporting the conclusions of this article will be made available by the authors, without undue reservation.

## Ethics Statement

Ethical review and approval was not required for the study on human participants in accordance with the local legislation and institutional requirements. Written informed consent for participation was not required for this study in accordance with the national legislation and the institutional requirements.

## Author Contributions

SG and DF collected and organized data. DF wrote the main body of text. HW, DF, DW, SG, and SR provided editing. All authors contributed to the article and approved the submitted version.

## Funding

This study received funding from Abbott Laboratories, Inc. The funder was not involved in the study design, collection, analysis, interpretation of data, the writing of this article or the decision to submit it for publication.

## Conflict of Interest

SR has received consultancy or speaker fees from Abbott Laboratories. DF has received consultancy or speaker fees from Abbott Laboratories, Abbvie, Amneal, Acorda, GE, Kyowa Kirin, Neurocrine, Sunovion, and also receives compensation as an FTC and Justice Department subject matter expert. The remaining authors declare that the research was conducted in the absence of any commercial or financial relationships that could be construed as a potential conflict of interest.

## Publisher's Note

All claims expressed in this article are solely those of the authors and do not necessarily represent those of their affiliated organizations, or those of the publisher, the editors and the reviewers. Any product that may be evaluated in this article, or claim that may be made by its manufacturer, is not guaranteed or endorsed by the publisher.
